# Anticentromere antibody positive Sjögren's Syndrome: a retrospective descriptive analysis

**DOI:** 10.1186/ar2958

**Published:** 2010-03-13

**Authors:** Vasiliki-Kalliopi K Bournia, Konstantina D Diamanti, Panayiotis G Vlachoyiannopoulos, Haralampos M Moutsopoulos

**Affiliations:** 1 Department of Pathophysiology, Medical School, National and Kapodistrian University, 75 Mikras Asias Street, 11527, Athens, Greece

## Abstract

**Introduction:**

A subgroup of patients with primary Sjögren's Syndrome (SS) and positive anticentromere antibodies (ACA) were recognized as having features intermediate between SS and systemic sclerosis (SSc). Our goal was to describe this group clinically and serologically and define its tendency to evolve to full blown SSc.

**Methods:**

Among 535 patients with primary SS we identified 20 ACA positive (ACA+/SS). We compared them to 61 randomly selected ACA negative SS patients (ACA-/SS), 31 ACA positive SSc patients with sicca manifestations [SSc/(+) sicca] and 20 ACA positive SSc patients without sicca manifestations [SSc/(-) sicca].

**Results:**

Prevalence of ACA among SS patients was 3.7%. Cases and controls did not differ in sex ratio and age at disease onset. ACA+/SS patients had a lower prevalence of dry eyes, hypergammaglobulinaemia, anti-Ro and anti-La antibodies and a higher prevalence of Raynaud's phenomenon and dysphagia compared to ACA-/SS patients. They also had lower prevalence of telangiectasias, puffy fingers, sclerodactyly, Raynaud's phenomenon, digital ulcers and gastroesophageal reflux in comparison to both of the SSc subgroups and a lower prevalence of dyspnoea and lung fibrosis compared to the SSc/(+) sicca subgroup. Two patients originally having ACA+/SS evolved to full blown SSc. Four deaths occurred, all among SSc patients. Kaplan Meier analysis showed a significant difference between cases and controls in time from disease onset to development of gastroesophageal reflux, telangiectasias, digital ulcers, arthritis, puffy fingers, xerostomia, hypergammaglobulinaemia and dysphagia.

**Conclusions:**

ACA+/SS has a clinical phenotype intermediate between ACA-/SS and SSc and shows little tendency to evolve to SSc.

## Introduction

Sjögren's Syndrome (SS) is a chronic autoimmune disease characterized by lymphocytic infiltration of the exocrine glands. It can present both with glandular and extraglandular manifestations [1,2] and may be either primary or associated with other rheumatic diseases. In approximately 60% of cases SS develops secondarily to other autoimmune conditions, most commonly rheumatoid arthritis, systemic lupus erythematosus or systemic sclerosis (SSc), while among those with various other systemic autoimmune diseases SS has a prevalence of 20% [1,3]. A subset of patients with primary disease, who present features intermediate between SS and limited cutaneous SSc has been previously recognized [4-6]. Their common characteristic is the finding of anticentromere antibodies (ACA) detected by immunofluorescence on Hep-2 cells. It remains to be answered whether these ACA positive SS patients represent merely a SS subgroup or if they constitute a transitional phase in the evolution to full blown SSc. Our goal was to clinically and immunologically characterize ACA positive SS patients in comparison to both ACA negative SS patients and ACA positive SSc patients, and to determine their tendency to evolve to definite SSc.

## Materials and methods

### Patients

We retrospectively studied the charts of 535 SS patients seen in our outpatient clinic between 1981 and 2009. Amongst them we identified 20 ACA positive patients (ACA+/SS), who fulfilled the American-European consensus criteria for the classification of SS [7]. Our control groups consisted of 61 subjects randomly selected from the pool of ACA negative SS patients (ACA-/SS) (1 out of every nine patients) and another 51 ACA positive SSc patients, divided in two subgroups depending on the presence (SSc/(+) sicca, n = 31) or absence (SSc/(-) sicca, n = 20) of concomitant sicca manifestations. Twelve SSc patients in the first subgroup fulfilled criteria for secondary SS according to the American European consensus group criteria. Diagnosis of SSc was based on the preliminary classification criteria of the American Rheumatism Association [8] and the criteria for classification of early SSc, proposed by LeRoy and Medsger in 2001 [9]. Patients satisfying criteria for prescleroderma or very early SSc, as recently put forward by the European League Against Rheumatism Scleroderma Trials and Research Group (EUSTAR) [10], were not included in the SSc group, since our main goal was to examine progression of ACA+/SS patients to definite SSc. The design of our study was approved by the Laikon Hospital Ethics Committee and written informed consent was obtained from all participants or from the first degree relatives of those deceased.

### Data collection

For every study participant we collected demographic, clinical and immunological data, both at first visit and cumulatively over the entire follow up period. Disease onset was defined by the appearance of Raynaud's Phenomenon, sicca manifestations, salivary gland enlargement, arthritis, purpura, puffy fingers, sclerodactyly, digital ulcers, calcinosis, dysphagia, gastroesophageal reflux, pulmonary arterial hypertension or lung fibrosis. Table 1 presents the first disease symptom by disease category. Abnormal findings in minor salivary gland biopsy, Schirmer test and Rose Bengal stain were defined as elsewhere described [7,11]. Tear film break up time of less than 10 seconds and subjective complaints of dry eyes, xerostomia, dyspnoea and dysphagia were additionally documented. Salivary gland enlargement, lymphadenopathy, arthritis, purpura, telangiectasias, puffy fingers, digital ulcers, Raynaud's phenomenon, calcinosis and sclerodactyly were recorded as assessed by the attending physician. Diagnosis of gastroesophageal reflux was based on clinical symptoms of heartburn and regurgitation, further confirmed by gastroscopy [12]. If a right heart catheterization had not been performed, the diagnosis of pulmonary arterial hypertension was made by heart Doppler echocardiography, using a pulmonary artery systolic pressure (PASP) = 40 mmHg as a cutoff, since values above this were shown in a SSc cohort to predict more accurately the finding of pulmonary arterial hypertension in cardiac catheterization [13]. Lung fibrosis was documented by chest x-ray and when needed by high resolution computed tomography, allowing identification of ground glass opacities. Serositis (pleuricy or pericarditis) was diagnosed radiologically or echocardiographically. Recognition of a restrictive pattern on pulmonary function tests required a reduction of total lung capacity below 75% of the predicted value, or the combination of a forced vital capacity (FVC) below 75% of the predicted value and a normal or above normal forced expiratory volume in first second (FEV1)/FVC ratio. Peripheral neuropathy was diagnosed by neurophysiologic testing. Carpal tunnel syndrome was documented based on the recommendations of the National Institute of Occupational Safety and Health [14]. Renal involvement was defined by the development of acute onset hypertension and elevated serum creatinine or impaired creatinine clearance (<60 ml/minute), elevated urinary pH (>6), proteinuria (>300 mg/day) or the presence of active urine sediment. Regarding laboratory tests, white blood cell count <4 K/μL, C3 <75 mg/dl, C4 <10 mg/dl, rheumatoid factor >20 I U/ml and γ-globulin >1.7 g/dl were considered abnormal. For the diagnosis of primary biliary cirrhosis and autoimmune hepatitis the criteria described elsewhere were used [15,16]. The diagnosis of celiac and atrophic gastritis required serological or histological evidence [17,18]. Hashimoto was diagnosed on the grounds of clinical or subclinical hypothyroidism and positive anti-thyroid peroxidase and/or anti-thyroglobulin antibodies [19]. Diagnosis of lymphoma required histological confirmation.

**Table 1:** First symptom by disease category.

First symptom	Disease category			
	
	ACA+/SS (n = 20) [%]	SSc/(-) sicca (n = 20) [%]	SSc/(+) sicca (n = 31) [%]	ACA-/SS (n = 61) [%]
Raynaud phenomenon	6 [30.00]	18 [90.00]	29 [93.55]	5 [8.20]
sicca manifestations	15 [75.00]	-	6 [19.35]	52 [85.25]
SGE	1 [5.00]	-	1 [3.23]	12 [19.67]
arthritis	1 [5.00]	1 [5.00]	2 [6.45]	4 [6.56]
purpura,	-	-	-	4 [6.56]
puffy fingers/sclerodactyly	1 [5.00]	6 [30.00]	8 [25.81]	4 [6.56]
digital ulcers	-	2 [10.00]	1 [3.23]	-
dysphagia/GER	1 [5.00]	1 [5.00]	1 [3.23]	-
lung fibrosis	-	-	-	1 [1.64]

Some patients reported more than one initial symptom. SGE, salivary gland enlargement, GER, gastroesophageal reflux

### Statistical analysis

We compared patients' characteristics between the ACA+/SS and each of the control groups, using Pearson's χ^2 ^test, Fisher's exact test and one way analysis of variance (ANOVA) with Bonferroni post hoc analysis, as indicated. Two-sided probability (*P*) values < 0.05 were considered statistically significant. Median time from disease onset to death or to development of particular clinical symptoms and laboratory findings was derived from Kaplan Meier curves and tested for statistical significance by the log-rank statistic. The Statistical Package for the Social Sciences (version 17, SPSS Inc., Chicago, Illinois, USA) was used for the analysis.

## Results

### Demographics, follow-up and disease duration of cases and controls

Of the 535 SS patients in our cohort, 20 were ACA positive, corresponding to a prevalence of 3.7%. All patients in this group were female, with an age at disease onset of 52.35 ± 3.35 years [mean ± standard error (SE)]), disease duration of 12.13 ± 1.65 years (mean ± SE) and a follow up of 5.38 ± 1.15 years (mean ± SE). As seen in Table 2, these variables did not differ significantly between cases and controls. Moreover, cases and controls did not differ with regard to sex ratio or smoking habits (Table 3).

**Table 2:** Age at disease onset, disease duration and duration of follow up of cases and controls

Disease	*Characteristics*					
	**Age at first symptom (*P *= 0.108)**^α^			Age at first non-Raynaud symptom (*P *= 0.279)^α^		
	*Mean*	*SD*	*Mean difference (P-value)*^*b*^	*Mean*	*SD*	*Mean difference (P-value)*^*b*^
**ACA+/SS (n = 20)**	52.35	15.0		53.55	15.2	
**SSc/(+) sicca (n = 31)**	43.29	15.8	0.187	47.23	13.2	0.699
**SSc/(-) sicca (n = 20)**	42.45	15.5	0.196	47.3	15.7	0.954
**ACA-/SS (n = 61)**	46.52	13.31	0.729	46.6	13.3	0.341
						
	**Disease duration from onset of first symptom **(*P *< 0.0001)^α^			**Disease duration from onset of first non-Raynaud symptom **(*P *= 0.036)^α^		

	*Mean*	*SD*	*Mean difference (P-value)*^*b*^	*Mean*	*SD*	*Mean difference (P-value)*^*b*^
**ACA+/SS (n = 20)**	12.13	7.4		10.96	7.0	
**SSc/(+) sicca (n = 31)**	17.51	11.1	0.248	13.48	6.7	1.000
**SSc/(-) sicca (n = 20)**	15.99	11.7	1.000	11.20	8.6	1.000
**ACA-/SS (n = 61)**	8.89	7.4	1.000	8.78	7.3	1.000
						
	**Duration of Follow up **(*P *= 0.012)^α^			-		

	*Mean*	*SD*	*Mean difference (P-value)*^*b*^	**-**		
**ACA+/SS (n = 20)**	5.38	5.1		**-**		
**SSc/(+) sicca (n = 31)**	7.87	5.2	0.494	**-**		
**SSc/(-) sicca (n = 20)**	4.00	4.1	1.000	**-**		
**ACA-/SS (n = 61)**	4.49	5.0	1.000	**-**		

a. overall *P *from ANOVA

b. Difference in mean when compared with group **ACA (+) SS**.

Data were calculated using both the first symptom and the first non Raynaud symptom as a starting point.

**Table 3:** Patients' demographics, autoimmune profile and co-morbidities by disease category

***Characteristics of patients (P-value***^1^)	ACA+/SS (n = 20) [%]	SSc/(+) sicca (n = 31) [%]	SSc/(-) sicca (n = 20) [%]	ACA-/SS (n = 61) [%]
**Sex**(0.558)				
*female*	20 [100.0]	28 [90.3]	19 [95.0]	57 [93.4]
*male*	0 [0.0]	3 [9.7]	1 [5.0]	4 [6.6]
*P-values*^2^		0.271	1.000	0.567
**Smoking **(0.603)	4 [20.0]	5 [16.1]	2 [10.0]	14 [23.0]
*P --values*^2^		0.703	0.661	1.000
**Anti-Ro (<0.0001)**	6 [30.0]	4 [12.9]	0 [0.0]	43 [70.5]
*P-values*^2^		0.163	**0.020**	**0.003**
**Anti-La **(**<0.0001**)	3 [15.0]	2 [6.5]	0 [0.0]	25 [41.0]
*P-values*^2^		0.369	0.231	**0.056**
**ANA (0.006)**	20 [100.0]	31 [100.0]	20 [100.0]	51 [83.6]
*P-values*^2^		-	**-**	0.060
**Anti-U1RNP **(0.558)	0 [0.0]	3 [9.7]	1 [5.0]	4 [6.6]
*P-values*^2^		0.271	1.000	0. 567
**Anti-Sm **(0.721)	0 [0.0]	1 [3.2]	0 [0.0]	2 [3.3]
*P-values*^2^		1.000	-	1.000
**Anti-Scl70 **(0.172)	0 [0.0]	2 [6.5]	1 [5.0]	0 [0.0]
*P-values*^2^		0.514	1.000	-
**Th. Hashimoto (0.023)**	1 [5.0]	4 [12.9]	0 [0.0]	15 [24.6]
*P-values*^2^		0.636	1.000	0.102
**Autoim. Hepatitis **(-)	0 [0.0]	0 [0.0]	0 [0.0]	0 [0.0]
*P-values*^2^		-	-	-
**Atr. gastritis **(0.422)	2 [10.0]	2 [6.5]	0 [0.0]	2 [3.3]
*P-values*^2^		0.640	0.487	0.254
**PBC **(0.209)	3 [15.0]	3 [9.7]	0 [0.0]	3 [4.9]
*P-values*^2^		0.668	0.231	0.157
**Celiac **(0.209)	0 [0.0]	0 [0.0]	0 [0.0]	1 [1.6]
*P-values*^2^		-	-	1.000
**Lymphoma **(0.476)	0 [0.0]	2 [6.5]	0 [0.0]	2 [3.3]
*P-values*^2^		0.514	-	1.000
**Death **(0.059)	0 [0.0]	3 [9.7]	1 [5.0]	0 [0.0]
*P-values*^2^		0.271	1.000	-

^1 ^Overall *P*-value from the Chi Square test of independence (significance < 0.05).

^2 ^*P*-values from the Fisher's exact test comparing the first group with each of the other three groups

PBC, primary biliary cirrhosis

### Immunological profile and histological findings of cases and controls

Anti-Ro/SS-A and anti-La/SS-B autoantibodies were detected in 30% and 15% of ACA+/SS patients, compared to 70.5% (*P *= 0.003) and 41% (*P *= 0.056) of ACA-/SS patients, respectively. The SSc/(+) sicca group did not differ significantly from the ACA+/SS group in the prevalence of anti-Ro/SS-A and anti-La/SS-B autoantibodies, while SSc/(-) sicca patients displayed neither anti-Ro/SS-A nor anti-La/SS-B reactivity in their sera. Other autoantibodies such as anti-U1RNP, anti-Sm and anti-Scl70 occurred rarely and their prevalence did not differ significantly between groups (Table 3).

Minor salivary gland biopsy had been performed in 19 ACA+/SS patients, 57 ACA-/SS patients and 16 SSc/(+) sicca patients, resulting in an equal frequency (94.7%) of abnormal findings in the two SS groups, and a somewhat lower frequency in the SSc/(+) sicca subgroup (75%), although this difference was not significant. Similarly, cases and controls undergoing ocular examination with Rose Bengal stain, Schirmer test and tear film break up time had a comparable prevalence of abnormal results (Table 4).

**Table 4:** Patients' clinical characteristics by disease category, cumulatively for the entire follow up period

*Characteristics of patients * ***(P-value***^1^)	ACA+/SS (n = 20) [%]	SSc/(+) sicca (n = 31) [%]	SSc/(-) sicca (n = 20) [%]	ACA-/SS (n = 61) [%]
**Positive MSGB (0.039)**	18 [94.7]	12 [75.0]	-	54 [94.7]
*P-values*^2^		0.156		1.000
**Abnormal Rose Bengal **(0.364)	8 [72.7]	9 [56.3]	0 [0.0]	22 [71.0]
*P-values*^2^		0.448	0.333	1.000
**Abnormal Schirmer test **(0.176)	9 [64.3]	14 [87.5]	0 [0.0]	35.5 [71.4]
*P-values*^2^		0.204	0.400	0.743
**Abnormal BUT **(0.428)	7 [77.8]	9 [75.0]	0 [0.0]	18 [69.2]
*P-values*^2^		1.000	0.300	1.000
**Lymphadenopathy **(0,249)	3 [15.0]	8 [25.8]	1 [5.0]	9 [14.8]
*P-values*^2^		0.493	0.605	1.000
**Raynaud (<0.0001)**	15 [75.0]	31 [100.0]	20 [100.0]	11 [18.0]
*P-values*^2^		**0.007**	**0.047**	**<0.0001**
**SGE (0.006)**	3 [15.0]	4 [12.9]	0 [0.0]	20 [32.8]
*P-values*^2^		1.000	0.231	0.160
**Purpura (0,052)**	2 [10.0]	1 [3.2]	0 [0.0]	11 [18.0]
*P-values*^2^		0.553	0.487	0.502
**Xerostomia (<0.0001)**	19 [95.0]	25 [80.6]	0 [0.0]	53 [86.9]
*P-values*^2^		0.223	**<0.0001**	0.440
**Dry eyes (<0.0001)**	17 [85.0]	29 [93.5]	0 [0.0]	60 [98.4]
*P-values*^2^		0.369	**<0.0001**	**0.045**
**Telangiectasias (<0.0001)**	2 [10.0]	28 [90.3]	14 [70.0]	2 [3.3]
*P-values*^2^		**<0.0001**	**<0.0001**	0.254
**Puffy fingers (<0.0001)**	7 [35.0]	26 [83.9]	14 [70.0]	10 [16.4]
*P-values*^2^		**0.001**	**0.056**	0.112
**Sclerodactyly (<0.0001)**	0 [0.0]	31 [100.0]	19 [95.0]	0 [0.0]
*P-values*^2^		**<0.0001**	**<0.0001**	-
**Calcinosis (0. 001)**	0 [0.0]	6 [19.4]	4 [20.0]	0 [0.0]
*P-values*^2^		0.070	0.106	**-**
**Ulcers **(0.249)	2 [10.0]	17 [54.8]	11 [55.0]	0 [0.0]
*P-values*^2^		**0.001**	**0.006**	**0.059**
**Dysphagia (<0.0001)**	7 [35.0]	18 [58.1]	5 [25.0]	4 [6.6]
*P-values*^2^		0.153	0.731	**0.004**
**GER (<0.0001)**	3 [15.0]	20 [64.5]	10 [50.0]	6 [9.8]
*P-values*^2^		**0.001**	**0.041**	0.682
**Arthritits **(0.372)	8 [35.0]	10 [32.3]	4 [20.0]	14 [23.0]
*P-values*^2^		0.765	0.301	0.156
**Dyspnoea (<0.0001)**	7 [40.0]	20 [64.5]	5 [25.0]	9 [14.8]
*P-values*^2^		**0.049**	0.731	0.060
**PAH (0.004)**	2 [10.0]	8 [25.8]	2 [10.0]	1 [1.6]
*P-values*^2^		0.280	1.000	0.149
**Ground glass HRCT **(0.606)	0 [0.0]	4 [21.1]	1 [9.1]	1 [12.5]
*P-values*^2^		0.544	1.000	1.000
**Lung fibrosis (0.001)**	2 [10.0]	12 [38.7]	7 [35.0]	5 [8.2]
*P-values*^2^		**0.029**	0.127	1.000
**Restrict. pattern PFTs **(0.103)	2 [22.2]	8 [30.8]	6 [42.9]	0 [0.0]
*P-values*^2^		1.000	0.400	0.211
**Serositis **(0.080)	3 [15.0]	6 [19.4]	3 [15.0]	2 [3.3]
*P-values*^2^		1.000	**0.019**	0.094
**Renal involvement **(0.760)	0 [0.0]	0 [0.0]	0 [0.0]	1 [1.6]
*P-values*^2^		**-**	**-**	1.000
**Carpal tunnel **(0.853)	2 [10.0]	3 [9.7]	3 [15.0]	5 [4.9]
*P-values*^2^		1.000	1.000	1.000
**Per. Neuropathy **(0.743)	1 [5.0]	2 [6.5]	0 [0.0]	3 [86.9]
*P-values*^2^		1.000	1.000	1.000
**Leucopenia **(0,088)	2 [10.0]	2 [6.5]	2 [10.0]	15 [24.6]
*P-values*^2^		0.640	1.000	0.216
**Hypergammaglobulinaemia (0.0001)**	5 [25.0]	6 [19.4]	1 [5.0]	31 [50.8]
*P-values*^2^		0.732	0.182	**0.068**
**Cryoglobulinaemia **(0.609)	0 [0.0]	2 [6.5]	1 [5.0]	5 [4.9]
*P-values*^2^		0.514	1.000	0.326
**RF (0.015)**	5 [25.0]	6 [19.4]	4 [20.0]	29 [47.5]
*P-values*^2^		0.732	1.000	0.117
**Low C3,C4 **(0.289)	0 [0.0]	5 [20.0]	3 [23.1]	10 [19.6]
*P-values*^2^		0.137	0.087	0.101
**Proteinuria **(0.760)	0 [0.0]	0 [0.0]	0 [0.0]	1 [1.6]
*P-values*^2^		**-**	**-**	1.000

^1 ^Overall *P*-value from the Chi Square test of independence (significance < 0.05).

^2 ^*P*-values from the Fisher's exact test comparing the first group with each of the other three groups MSGB: minor salivary gland biopsy, BUT, Tear film break up time, SGE, salivary gland enlargement, GER, gastroesophageal reflux, PAH, pulmonary arterial hypertension, HRCT, high resolution computed tomography, PFT, pulmonary function tests, RF, rheumatoid factor

### Clinical and laboratory features of cases and controls

Differences in the prevalence of clinical and laboratory characteristics of cases and controls were detected already at first visit and, as a general rule, they persisted or became even more pronounced, when the entire follow up period was considered. More specifically for the entire follow up period, telangiectasias, puffy fingers, sclerodactyly, Raynaud's phenomenon, digital ulcers and gastroesophageal reflux were significantly less frequent among ACA+/SS patients as compared to both SSc subgroups. The ACA+/SS group also had a lower prevalence of dyspnoea (*P *= 0.049) and lung fibrosis (*P *= 0.029) in comparison to the SSc/(+) sicca group. Compared to ACA-/SS, ACA+/SS patients were less prone to develop dry eyes (*P *= 0.045), but more inclined to develop Raynaud's phenomenon (*P *= 0.0001) or dysphagia (*P *= 0.004). Dyspnoea and digital ulcers were also more prominent in the ACA+/SS group, but not at a statistically significant level (Table 4).

Among those having undergone a pulmonary function test, a restrictive pattern was seen in 22.2% of ACA+/SS patients as compared to 30.8% of SSc/(+) sicca patients (*P *= 1.000), 42.9% of SSc/(-) sicca patients (*P *= 0.400) and none of the ACA-/SS patients (*P *= 0.211). Only a small proportion of patients in each group had done a chest high resolution computed tomography, on the basis of a previous chest x-ray marginally indicative of an interstitial pattern. Although small numbers do not allow for safe conclusions, no statistically significant difference was seen in the frequency of ground glass pattern between ACA+/SS patients and controls.

When comparing cases to controls with reference to laboratory findings, no statistically significant differences were observed, with the exception of hypergammaglobulinaemia, which tended to be more frequent in the ACA-/SS compared to the ACA+/SS group (*P *= 0.068). None of the patients in any of the groups had proteinuria (Table 4).

### Prevalence of co-morbidities in cases and controls

Thyroiditis Hashimoto affected 5% of ACA+/SS patients, compared to 24.6% of ACA-/SS patients (*P *= 0.102) and 12.9% of SSc/(+) sicca patients (*P *= 0.636). For primary biliary cirrhosis (PBC) the respective proportions were 15%, as compared to 4.9% (*P *= 0.157) and 9.7% (*P *= 0.668), while the prevalence for atrophic gastritis was 10%, compared to 3.3% (*P *= 0.254) and 6.5% (*P *= 0.640) respectively. None of the SSc/(-) sicca patients had any of the above conditions. Differences between cases and controls were non-significant. Autoimmune hepatitis did not occur in any of the groups and only one patient in the ACA-/SS group had celiac (Table 3).

### Evolution of ACA+/SS patients

During our follow up period two patients in the ACA-/SS group and another two in the SSc/(+) sicca subgroup developed lymphoma, while four patients died, all of which had SSc. Differences in mortality and lymphoma development between cases and controls were not significant (Table 3).

The overwhelming majority of ACA positive SSc/(+) sicca patients fulfilled criteria for SSc, already at their first visit. Of the 31 patients in this group, only two started out as ACA+/SS to which a diagnosis of SSc was added, in both cases after one year of follow up. One of these two patients later developed pulmonary arterial hypertension and died. Another three patients with Raynaud's phenomenon, puffy fingers, ACA and sicca manifestations at first visit eventually developed SSc, after 3, 9 and 24 months respectively, but never fulfilled criteria for SS.

### Time to development of particular symptoms

**Figure 1:**
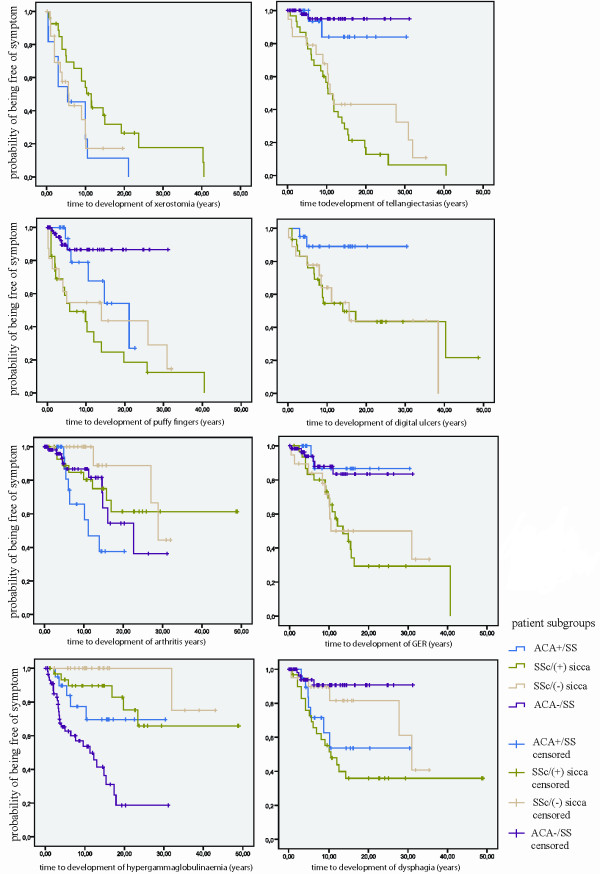
**Kaplan Meier analysis**. Kaplan Meier curves for time to development of Gastroesophageal reflux (GER), dysphagia, arthritis, telangiectasias, digital ulcers, puffy fingers, xerostomia, and hypergammaglobulinaemia. The X axis depicts time in years from the presentation of the first symptom.

Distribution of time from disease onset to development of certain clinical features was examined for the four groups of patients. In comparison to the ACA+/SS group, telangiectasias, digital ulcers and gastroesophageal reflux tended to occur sooner in the SSc subgroups, finally affecting these groups almost entirely. Puffy fingers developed sooner and xerostomia developed later in the SSc/(+) sicca compared to the ACA+SS group. Regarding development of arthritis, it occurred later in the SSc/(-) sicca subgroup compared to the ACA+/SS group. Likewise, when comparing ACA+/SS to ACA-/SS patients, dysphagia appeared earlier in the first group while hypergammaglobulinaemia in the second group. Statistics for survival curves depicting time to death or to development of sclerodactyly and calcinosis could not be calculated since all cases in the ACA+/SS and ACA-/SS groups were censored (Figure 1).

## Discussion

In an earlier, retrospective, study of 41 ACA positive patients a prevalence of 17% was reported for primary SS, thus for the first time establishing an association between ACA and primary SS [6]. This finding was corroborated by other investigators who showed that the prevalence of primary SS in different ACA positive cohorts ranged from 2.5% to 12% [4,20-24]. Our present goal was to focus on the clinical and serological characteristics and on the outcome of ACA/+SS patients, after a long follow up.

In our study, ACA were found in 3.7% of patients with SS, which is in agreement with some reports estimating this prevalence in the range of 4.5% to 8.7% [5,25], but seems somewhat lower compared to the percentages reported by Katano et al (24.6%) [26], Chan et al. (14.8%) [24] or Caramaschi et al. (16.6%) [27].

In accordance to previous studies [5,26,28] all of our groups demonstrated a striking female preponderance. Our findings, however, do not corroborate those of other investigators [5,26,28], reporting a higher mean age at disease onset in the ACA+/SS, compared to the ACA/-SS group.

We and other researchers [5,6,27,28] have shown a significantly lower prevalence of anti-Ro/SS-A and anti-La/SS-B autoantibodies among ACA+/SS compared to ACA-/SS patients. As expected, anti-Ro/SS-A and anti-La/SS-B autoantibodies were absent in the SSc/(-) sicca subgroup. However, their prevalence in the SSc/(+) sicca subgroup was similar to that found in the ACA+/SS group, indicating that, with respect to immunological profile, ACA+/SS displays features of an overlap between SS and SSc. Contrary to others [27-29], we didn't find significantly lower rates of rheumatoid factor positivity in the ACA+/SS compared to the ACA-/SS group. In addition, previous reports have detected autoantibodies other than ACA, anti-Ro/SS-A, anti-La/SS-B and rheumatoid factor in more than 50% of ACA+/SS patients [5], whereas in our study none of the patients in this group presented anti-Scl 70, anti-U1RNP or anti-Sm autoantibodies. As shown in a previous publication, anti-Sm and anti-U1RNP are rare in Greek patients, even among those who suffer from systemic lupus erythematosus [30].

An association of ACA positive SS with primary biliary cirrhosis has been established in previous works [5], but was not seen in our cohort. Organ specific autoimmune diseases affecting our group of ACA+/SS patients encompassed primary biliary cirrhosis (15%), Hashimoto thyroiditis (5%) and atrophic gastritis (10%), but differences in prevalence between this group and the controls were not significant.

Our findings corroborate previous works that report a greater frequency of Raynaud's phenomenon [5,26,27] and a significantly lower frequency of hypergammaglobulinaemia [27,28] in patients with ACA+/SS compared to patients with ACA-/SS. However, in contrast to Katano et al. [26] and Caramashi et al. [27] we did not observe a lower prevalence of leucocytopenia in our group of ACA+/SS and found no difference in the frequency of peripheral neuropathy between cases and ACA-/SS controls, although contradictory reports exist on this issue [5,28].

Given that patients are likely to develop symptoms compatible with a lcSSc diagnosis not all at once, but gradually over time [31] there is a probability that ACA+/SS patients will eventually develop SSc in the long run. However, the lower prevalence we found for calcinosis, Raynaud's phenomenon, esophageal dysmotility, sclerodactyly, telangiectasias, puffy fingers, digital ulcers and lung fibrosis in the ACA+/SS group compared to the SSc subgroups, combined with an equal follow up duration of cases and controls, makes this evolutionary pattern unlikely. Only two patients that originally started out as ACA+/SS, developed symptoms compatible with a SSc diagnosis later during their follow up. In contrast, the majority of ACA+/SS patients retain during the course of their disease a clinical pattern distinct from both the ACA-/SS and the SSc groups.

There are previous reports of ACA+/SS patients having evolved to SSc. Caramashi et al[27] described four such cases out of a total of ten and Ramos-Casals et al. [25] additionally reported another three out of an initial group of eight. In the study performed by Salliot et al. [5] none of the 10 ACA+/SS patients advanced to lcSSc, while according to Miyawaki et al. [4] their cohort of 84 ACA positive SSc patients included six patients, who originally had primary SS and Raynaud's phenomenon before developing lcSSc. In total, counting in the two cases from our cohort, 15 out of 66 patients (23%) initially presenting with ACA+/SS eventually developed SSc.

Patients with SS are at a higher risk than the general population for lymphoma development, demonstrating a standardized incidence rate of 18.9 (95% CI (9.4 to 37.9)) [32,33]. Histologically, the mucosa associated lymphoid tissue (MALT) subtype of non-Hodgkin lymphomas is the most commonly found. Some of the major risk factors for lymphoma development in SS patients include parotid gland enlargement, purpura, hypocomplementemia and cryoglobulinemia [33]. Despite the fact that in a recently published case report the presence of ACA antibodies in two pSS patients was associated with an increased risk for small vessel cutaneous vasculitis, parotid enlargement, low C4 complement levels, positive rheumatoid factor and lymphoma [34], none of these associations were corroborated by our study. On the contrary and in agreement with a previous report [27], none of the ACA+/SS patients in our cohort developed lymphoma. In addition, differences between cases and controls with respect to mortality or development of lymphoma were not significant.

Previous studies did not define a *gold standard *to effectively predict whether an ACA+/SS patient will retain the same disease pattern in the future. It seems that only by long term follow up can this question be answered. Therefore the time to development of a symptom could form a basis on which to decide if the clinical course of ACA positive SS will remain stable. By means of the Kaplan Meier survival analysis we identified an earlier development of symptoms compatible with the diagnosis of SSc in one or both of the SSc subgroups, as compared to the ACA+/SS group. This could indicate that SSc patients acquire their disease phenotype rather soon, whereas ACA+/SS patients tend to keep a stable clinical pattern.

A weakness in our study was the retrospective design. In addition, we cannot rule out the possibility that some of the differences observed between the patient and control groups were due to statistical error type I, caused by multiple comparisons. Furthermore, our control group of SSc patients with sicca manifestations might not be homogenous, in the sense that it includes patients with secondary SS, but also patients for which sicca symptoms could possibly be attributed to the presence of glandular fibrosis [35]. Finally in some cases the number of patients censored was too big to allow for safe conclusions to be drawn from the Kaplan-Meier statistics.

## Conclusions

In conclusion, we have found a lower frequency of dry eyes, hypergammaglobulinaemia, anti-Ro/SS-A and anti-La/SS-B autoantibodies and a higher frequency of Raynaud's phenomenon and dysphagia in ACA+/SS as compared to ACA-/SS patients. A lower frequency of calcinosis, Raynaud's phenomenon, esophageal dysmotility, sclerodactyly and telangiectasias was also found in the ACA+/SS group in comparison to ACA positive SSc patients, both with and without sicca manifestations. In addition, only two patients in our cohort and less than one quarter of the ACA+/SS patients described in the literature eventually advanced to SSc, despite a long follow-up period. Our findings corroborate the results of previous studies reporting that ACA+/SS patients present clinical features intermediate between ACA negative SS and SSc, while indicating that these patients, in their majority, tend not to evolve to full blown SSc.

## Abbreviations

ACA-/SS: anticentromere antibody negative Sjögren syndrome; ACA: anticentromere antibodies; ACA+/SS: anticentromere antibody positive Sjögren syndrome; ANOVA: analysis of variance; FEV1: forced expiratory volume in first second; FVC: forced vital capacity; MALT: mucosa associated lymphoid tissue; PASP: pulmonary artery systolic pressure; SE: standard error; SS: Sjögren syndrome; SSc/(-) sicca: anticentromere antibody positive systemic sclerosis without sicca manifestations; SSc/(+) sicca: anticentromere antibody positive systemic sclerosis with sicca manifestations; SSc: systemic sclerosis

## Competing interests

The authors declare that they have no competing interests.

## Authors' contributions

VKB participated in the data collection, performed the statistical analysis and helped to draft the manuscript. KD participated in the data collection. PGV participated in the design and coordination of the study and in the drafting and critical revision of the manuscript. HMM conceived of the study, participated in its design and coordination and critically revised the manuscript. All authors read and approved the final manuscript.
